# Supplementary material for: Clinical Effect of Lenvatinib Re-Administration after Transcatheter Arterial Chemoembolization in Patients with Intermediate Stage Hepatocellular Carcinoma
Source: Cancers (Basel). 2022 Dec 13;14(24):6139. doi: 10.3390/cancers14246139 (PMC9776720; doi:10.3390/cancers14246139)
Supplement: Supplementary file 1 [file cancers-14-06139-s001.zip › cancers-2057195-supplementary.pdf]

*Article*

# Supplementary materials: Clinical effect of lenvatinib re-administration after transcatheter arterial chemoembolization in patients with intermediate stage hepatocellular carcinoma

**Seiichi Mawatari, Tsutomu Tamai, Kotaro Kumagai, Akiko Saisyoji, Kaori Muromachi, Ai Toyodome, Ohki Taniyama, Haruka Sakae, Sho Ijuin, Kazuaki Tabu, Kohei Oda, Yasunari Hiramine, Akihiro Moriuchi, Kazuhiro Sakurai, Shuji Kanmura, Akio Ido**

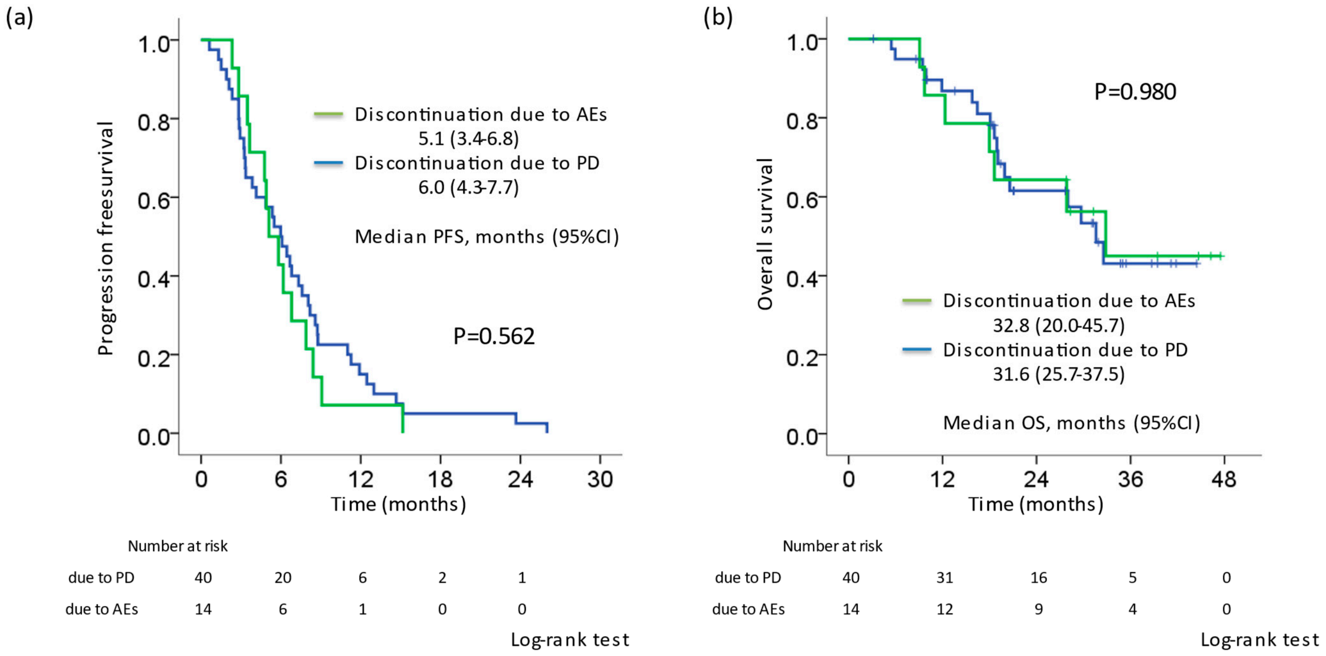


Figure S1: Kaplan–Meier curve of the overall survival (OS) and progression-free survival (PFS) according to the reasons for discontinuation. (a) The PFS from the first initiation of LEN. (b) The OS from the first initiation of LEN. There was no significant difference between the rate of dis-continuation due to AEs and that due to PD.
LEN, lenvatinib; AEs, adverse events; PD, progression disease;

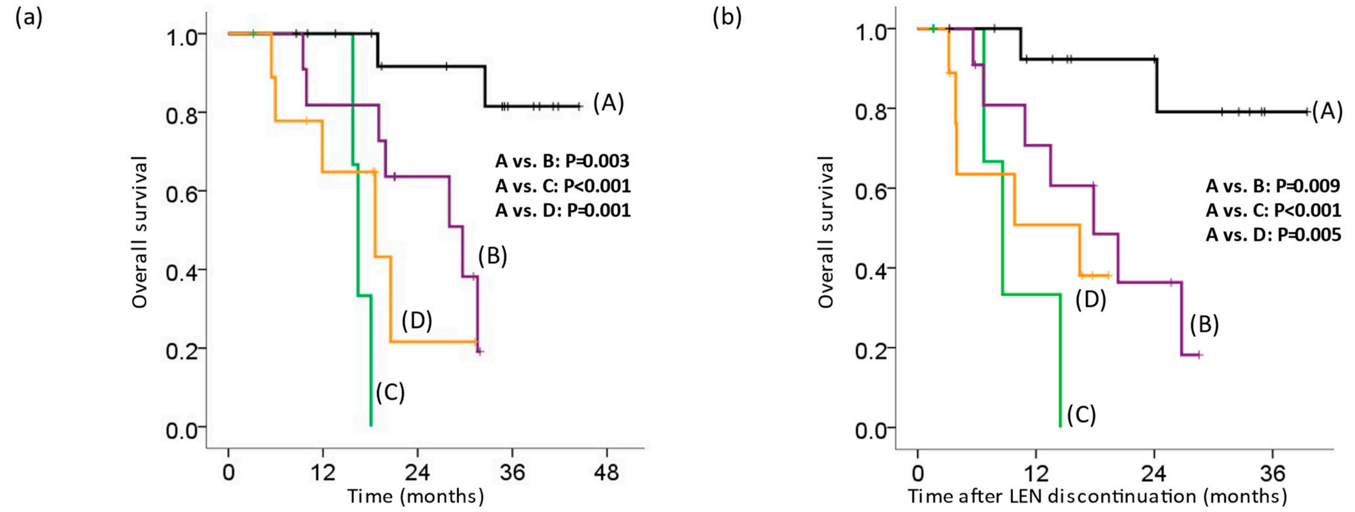


(a) Number at risk

| | | | | | |
|---|---|---|---|---|---|
| A: TACE-LEN | 16 | 14 | 10 | 5 | 0 |
| B: TACE-other therapy | 11 | 9 | 5 | 0 | 0 |
| C: Only TACE | 4 | 3 | 0 | 0 | 0 |
| D: Other therapy | 9 | 5 | 1 | 0 | 0 |

Log-rank test

(b) Number at risk

| | | | | |
|---|---|---|---|---|
| A: TACE-LEN | 16 | 11 | 8 | 1 |
| B: TACE-other therapy | 11 | 7 | 3 | 0 |
| C: Only TACE | 4 | 1 | 0 | 0 |
| D: Other therapy | 9 | 4 | 0 | 0 |

Log-rank test

Figure S2: Kaplan–Meier curve of the overall survival (OS) according to post-treatment in cases in which LEN was discontinued due to disease progression. (a) The OS from the first initiation of LEN. The Kaplan–Meier curve for the OS showed that group A had a better prognosis than groups B, C, and D. (b) The OS from the discontinuation of LEN. The Kaplan–Meier curve for the OS showed that group A had a better prognosis than groups B, C, and D.

LEN, lenvatinib;

Table S1. The reason of discontinuation of Lenvatinib

| | **ALL n=85** | **A: TACE-LEN n=19** | **B: TACE-other therapy* n=19** | **C : only TACE n=13** | **D: Other therapy* n=11** | **E: resection, RFA n=3** | **F: none n=20** | **P value** |
|---|---|---|---|---|---|---|---|---|
| disease progression | 55 | 17 | 13 | 7 | 10 | 1 | 7 | 0.073** |
| Adverse events | 29 | 2 | 6 | 6 | 1 | 2 | 12 | |
| deterioration of liver function | 8 | 0 | 1 | 1 | 0 | 0 | 6 | |
| gastrointestinal symptoms (including gastrointestinal bleeding) | 8 | 0 | 1 | 4 | 1 | 0 | 2 | |
| general malaise | 4 | 1 | 2 | 0 | 0 | 0 | 1 | |
| respiratory disease | 3 | 0 | 1 | 1 | 0 | 1 | 0 | |
| Others*** | 6 | 1 | 1 | 0 | 0 | 1 | 3 | |

One case was discontinued due to achieving CR.

*Other therapies such as Sorafenib, regorafenib, ramucirumab, cabozantinib, and atezolizumab + bevacizumab.

**A. vs. B, C, and D. (non-TACE-LEN).

*** portal vein thrombus (n=1), cerebral hemorrhage (n=1), and thrombocytopenia (n=1), and the exacerbation of pre-existing illness (n=3).

Table S2. Characteristics of post-treatment in patients for whom lenvatinib therapy was discontinued

| Characteristics | A：TACE-LEN n=18 | B：TACE-other therapy* n=17 | C：only TACE n=9 | D ： Other therapy* n=10 | P value |
|---|---|---|---|---|---|
| **Age, years** | 71.0±9.2 | 71.3±7.8 | 71.9±11.6 | 73.3±11.2 | 0.760 |
| **Sex, Male/Female** | 13/5 | 13/4 | 9/0 | 7/3 | 0.353 |
| **mALBI before therapy, 1/ 2a/ 2b/ 3** | 8/ 4/ 6/ 0 | 7/ 5/ 5/ 0 | 2/ 1/ 6/ 0 | 2/ 5/ 3/ 0 | 0.303 |
| **mALBI at EOT, 1/ 2a/ 2b/ 3** | 4/ 5/ 9/ 0 | 5/ 6/ 5/ 1 | 3/ 2/ 3/ 1 | 5/ 3/ 1/ 1 | 0.631 |
| **Maximum tumor diameter, mm** | 44.3±31.5 | 38.5±18.9 | 39.8±27.7 | 35.2±14.5 | 0.980 |
| **Number of tumors 0-3/ 4-6/ 7-** | 3/ 10/ 5 | 6/ 7/ 4 | 0/ 4/ 5 | 1/ 5/ 4 | 0.302 |
| **Up-to-seven in/out** | 4/ 14 | 4/ 13 | 1/ 8 | 2/ 8 | 0.892 |
| **Number of TACE treatments before LEN initiation, times** | 2.3±2.0 | 3.4±2.9 | 4.4±3.2 | 4.2±3.5 | 0.149 |
| **Platelet count ($\times 10^4/\mu L$)** | 12.8±5.2 | 16.3±6.2 | 19.5±11.0 | 16.9±5.0 | 0.107 |
| **AFP (ng/mL)** | 181±444 | 117±300 | 95±223 | 350±673 | 0.475 |
| **DCP (mAU/mL)** | 2263±7345 | 510±966 | 346±541 | 503±749 | 0.904 |
| **Start with a reduced amount, n (%)** | 9 (50.0) | 7 (41.2) | 7 (77.8) | 4 (40.0) | 0.295 |
| **Duration of LEN administration, days (post therapy)** | 236.8±148.1 (331.9±257.6) | 171.1±116.9 | 131.0±104.7 | 147.9±114.7 | 0.081 |
| **ORR, %** | 61.1 | 35.3 | 11.1 | 40.0 | 0.087 |
| **DCR, %** | 94.4 | 94.1 | 66.7 | 80.0 | 0.146 |
| **Discontinuation due to adverse events, %** | 11.1 | 35.3 | 55.6 | 10.0 | 0.041 |
| **Use of atezolizumab + bevacizumab, n (%)** | 8 (44.4) | 13 (76.5) | 0 (0) | 5 (50.0) | 0.003 |

Mean ± standard deviation

*Other therapies such as sorafenib, regorafenib, ramucirumab, cabozantinib, and atezolizumab + bevacizumab.

TACE, transcatheter arterial chemoembolization; LEN, lenvatinib; RFA, radiofrequency ablation; mALBI, modified albumin bilirubin score; AFP, alfa-fetoprotein; DCP, des-gamma carboxyprothrombin; ORR, objective response rate; DCR, disease control rate.
